# IVF outcome is optimized when embryos are replaced between 5 and 15 mm from the fundal endometrial surface: a prospective analysis on 1184 IVF cycles

**DOI:** 10.1186/1477-7827-11-114

**Published:** 2013-12-16

**Authors:** Valentina Rovei, Paola Dalmasso, Gianluca Gennarelli, Teresa Lantieri, Gemma Basso, Chiara Benedetto, Alberto Revelli

**Affiliations:** 1 Physiopatology of Reproduction and IVF Unit, Department of Surgical Sciences, University of Torino, Sant’Anna Hospital, Torino, Italy; 2 Medical Statistics Unit, Department of Public Health and Paediatrics, University of Torino, Torino, Italy

**Keywords:** Embryo transfer, US-guided embryo transfer, Implantation rate, Pregnancy rate, Abortion rate, Ongoing pregnancy rate

## Abstract

**Background:**

Some data suggest that the results of human in vitro fertilization (IVF) may be affected by the site of the uterine cavity where embryos are released. It is not yet clear if there is an optimal range of embryo-fundus distance (EFD) within which embryos should be transferred to optimize IVF outcome.

**Methods:**

The present study included 1184 patients undergoing a blind, clinical-touch ET of 1–2 fresh embryos loaded in a soft catheter with a low amount of culture medium. We measured the EFD using transvaginal US performed immediately after ET, with the aim to assess (a) if EFD affects pregnancy and implantation rates, and (b) if an optimal EFD range can be identified.

**Results:**

Despite comparable patients’ clinical characteristics, embryo morphological quality, and endometrial thickness, an EFD between 5 and 15 mm allowed to obtain significantly higher pregnancy and implantation rates than an EFD above 15 mm. The abortion rate was much higher (although not significantly) when EFD was below 5 mm than when it was between 5 and 15 mm. Combined together, these results produced an overall higher ongoing pregnancy rate in the group of patients whose embryos were released between 5 and 15 mm from the fundal endometrial surface.

**Conclusions:**

The site at which embryos are released affects IVF outcome and an optimal EFD range exists; this observations suggest that US-guided ET could be advantageous vs. clinical-touch ET, as it allows to be more accurate in releasing embryos within the optimal EFD range.

## Background

The technique used to perform embryo transfer (ET) is considered one of the most relevant factors affecting the final outcome of human in vitro fertilization (IVF) [1]. The type of catheter, the operator’s skill, the amount of loaded medium, the presence of mucus or blood in or around the catheter, any difficulty in entering the uterine cavity have all been recognized as variables affecting the IVF success rate [2,3].

One of the variables of ET technique potentially affecting IVF results could be the site of the uterine cavity at which embryos are released; despite some studies have been performed [4-10], it is not yet clear if there is an optimal site at which embryo deliver would give the best implantation rate. No conclusive evidence has been obtained even when, more recently, the site of the US-detectable air bubble was studied as a marker of the site of embryo release [11,12].

When ET is performed without any ultrasound guidance (clinical-touch ET, CTET), the site at which embryos are injected is rather variable, depending on the size of the uterus of each given patient; differently, ultrasound (US)-guided ET (USET) allows to deliver embryos wherever the operator wishes. Interestingly enough, although USET is obviously much more precise than CTET in transferring embryos at a pre-determined site of the uterine cavity, it has not been proven superior to CTET as far as the implantation and pregnancy rates are concerned [13].

To date, the optimal distance from the site of embryo transfer to the fundal endometrial surface (embryo-fundus distance, EFD) has not yet been identified, and also an EFD range out of which the chance of pregnancy is lowered has not been determined.

In the present study, we measured by transvaginal US the distance from the air bubble injected with the embryos just after CTET and the fundal endometrial surface (EFD) in order to assess 1) if EFD affects pregnancy and implantation rates, and 2) if there is a range of EFD within which embryos should be delivered to optimize IVF results, and out of which IVF outcome is significantly poorer.

## Methods

### Patients

The study involved patients undergoing their first IVF cycle with fresh embryo transfer at our IVF Unit between January 2010 and December 2012. It was approved by the local Ethical Committee (register number 35218/CE/A.210) and all patients gave their written informed consent.

Patients were enrolled with the aim to study a large, homogeneous group and minimize the incidence of factors potentially affecting embryo transfer and IVF outcome. Thus, the criteria used to exclude patients from the study were the following: 1) presence of an abnormal uterine cavity due to endometrial polyps, subcutaneous or intramural myomas distorting the uterine cavity, Műllerian malformations, endometrial synaechiae, etc. (assessed by transvaginal US and/or hysteroscopy); 2) presence of any systemic disease potentially reducing implantation rate (e.g. autoimmune diseases); 3) controlled ovarian hyperstimulation (COH) with any protocol other than the GnRH-agonist “long” protocol; 3) presence of an endometrial thickness ≤ 6 mm at the time of ET; 4) ET scheduled in any day other than day 2; 5) patients that required the change of catheter and the use of a stiffer catheter for cervical stenosis.

Finally, 1184 patients whose ET was scheduled on day 2 with the soft Sydney-Cook catheter, with endometrial thickness > 6 mm, who received one or two embryos (as routinely done in our IVF Unit), were included in the analysis. The basal clinical characteristics of these patients are reported in Table 1.

**Table 1:** Patients’ baseline characteristics

	**All**	**EFD (mm)**				**p**
		**<5**	**5-9.9**	**10-15**	**>15**	
	**(n = 1184)**	**(n = 59)**	**(n = 548)**	**(n = 429)**	**(n = 148)**	
***Age (years)***	35.2 ± 4.4	34.8 ± 4.2	35.2 ± 4.3	35 ± 4.7	35.8 ± 3.8	ns
***Smoking habit (%)***	16.4	16.4	18.6	15.3	12	ns
***BMI***	22.8 ± 3.4	24 ± 3.6	22.6 ± 3.2	22.8 ± 3.4	23 ± 3.6	ns
***BMI > 25 (%)***	23.8	22.3	20.9	23.1	29.9	ns
***Duration of infertility (years)***	3.8 ± 2.7	3.6 ± 2.2	3.8 ± 2.6	4 ± 2.8	3.8 ± 2.6	ns
***Main cause of infertility (%)***						
*Unexplained*	45.2	58.3	45.5	44.2	41.5	ns
*Severe endometriosis*	14	16.7	12	16.1	14.6	ns
*Reduced ovarian reserve*	17.9	15.6	21	15.8	16.9	ns
*Tubal factor*	22.9	19.4	21.5	23.9	27	ns
***Associated male factor (%)***	69.2	71.2	71.3	65.5	71.6	ns
***Basal FSH (UI/L)***	7.6 ± 3.1	6.8 ± 2.4	7.9 ± 3.2	7.5 ± 2.8	7.5 ± 3.5	ns
***Antral follicle count (AFC)***	15.3 ± 8.5	18.2 ± 10.9	15.1 ± 8.8	15.2 ± 8.1	15 ± 7.8	ns

Patients were subgrouped according to the site at which embryos were transferred (EFD = distance between the hyperecogenic spot and the basal layer of the endometrial fundus).

### IVF procedure

COH was performed using daily subcutaneous injections of rFSH (Follitropin alfa, Merck-Serono, Switzerland or Follitropin beta, MSD, Germany) or hMG (Meropur, Ferring, Germany) at appropriate doses (100–450 IU), estimated considering the woman’s age, the antral follicle count (AFC), and the basal (day 3) FSH circulating level. The long GnRH-agonist Buserelin (Hoechst, Germany, 0.3 mg intra-nasally three times a day) was used to achieve down-regulation in all IVF cycles included in the study.

Ovarian response to COH was monitored by transvaginal US plus serum estradiol (E2) measurement every third day from stimulation day 7. Ovulation was triggered by injecting subcutaneously 10,000 IU of hCG (Gonasi HP, IBSA, Switzerland) when at least two leading follicles reached 18 mm with appropriate serum E2 levels. Transvaginal US-guided oocyte aspiration (OPU) was performed approximately 36 hours after hCG injection under local anesthesia (paracervical block).

Either IVF or ICSI was performed according to the clinical indication, and after 2 days culture, one or two embryos were morphologically selected to be transferred in utero according to the previously published morphological score of Holte et al. [14]. Transvaginal progesterone (Crinone 8, Merck-Serono, Switzerland, 180 mg) was given daily for luteal phase support for 2 weeks, starting the day of embryo transfer.

### ET technique and assessment of the embryo-fundus distance (EFD)

All ETs were performed by the blind CTET technique using the soft Cook catheter (Cook, Australia). The catheter was loaded with 1–2 embryos suspended in 20 μl of culture medium; a 10 μl air bubble was loaded with the embryos in order to obtain an US-detectable marker. Embryo deliver was performed after inserting the internal soft catheter into the external guide and pushing it up to the third mark appearing on the internal catheter surface; this procedure allows the internal catheter tip to be placed exactly 3 cm beyond the bulb, in turn localized at the internal uterine os. After delivering the embryos, the internal catheter was retracted very slowly in order to minimize the risk of displacing the embryos from the original injection site. All ETs included in the study were performed by four experienced gynecologists whose work in the previous five years at the IVF Unit showed no operator-related differences in IVF outcome.

A few seconds after having completely retracted the transfer catheter, transvaginal US was performed to measure the distance between the air bubble (hyperechogenic spot) and the fundal endometrial surface (embryo-fundus distance, EFD). The US machine used in all cases and by all doctors performing the ET was an Aloka SSD-1700. In about 10% of cases, the air bubble was observed to slowly move from the injection site for about 30–60 seconds, probably as a consequence of slight uterine contractions; when it finally stopped acquiring a stable position, its final site was recorded for further analysis. The bubble displacement from the initial position never exceeded 15 mm and practically in all cases was directed backward, toward the cervix. A stable position was acquired within 60 seconds in all cases. The 1184 CTETs included in the study were classified into four subgroups according to the EFD: a) EFD <5 mm (n = 59); b) EFD between 5 and 9.9 mm (n = 548); c) EFD between 10 and 15 mm(n = 429); d) EFD >15 mm (n = 148).

### Outcomes

The primary outcome of the study was the clinical pregnancy rate (CPR)/ET, calculated as the ratio between the number of cases in which at least one gestational sac was seen at transvaginal US four weeks after ET and the number of performed ETs.

Secondary outcomes were the implantation rate (ratio between the number of gestational sacs and the number of transferred embryos; IR), the abortion rate (ratio between the number of pregnancies ended before 10 weeks and the number of clinical pregnancies; AR), and the ongoing pregnancy rate (ratio between the number of ongoing pregnancies after 10 weeks and the number clinical pregnancies; OPR).

The analysis of the outcomes was accomplished alsoafter subdividing patients according to the fertilization procedure (IVF or ICSI), and to the number of transferred embryos, one (single embryo transfer; SET) or two (double embryo transfer; DET).

### Statistical analysis

Data were expressed as mean ± SD or counts and percentages. Qualitative data were analyzed by means of Chi-square or Fisher’s exact test. The normality assumption of the quantitative measures was verified by Shapiro-Wilk test and significance of between-group differences were assessed using ANOVA or Kruskal Wallis rank test, as appropriate. Pairwise comparisons of the groups were performed with Bonferroni’s adjustment for multiple comparisons.

Generalized linear models (GLM) using logit link and binomial variance function were performed to assess the significance of the relationship between the 4 EFD subgroups and the different outcomes (pregnancy, implantation and abortion rates). Three models were fitted for each outcome: one model adjusting for the number of embryos transferred (1 or 2) and the type of procedure (IVF or ICSI), and two models stratifying on the number of embryos transferred and adjusting for procedure.

## Results

The 1184 patients included in the analysis had comparable clinical characteristics, and no significant differences among subgroups could be noticed as far as age, smoking habit, BMI, infertility duration, main infertility cause, and indexes of ovarian follicular reserve (basal FSH and antral follicle count; AFC), were concerned (Table 1).

The outcome of COH (total gonadotropin dose, number of retrieved oocytes, endometrial thickness) was similar in the four subgroups, and finally the mean number of transferred embryos did not differ among subgroups (Table 2). The morphological embryo score according to the score of Holte et al. [14] was not significantly different among subgroups both as for the mean score, and for the proportion of top-scored (10 points) embryos (Table 2).

**Table 2:** Controlled ovarian stimulation, embryological data and IVF outcome

	**All**	**EFD (mm)**				**p**
		**<5**	**5-9.9**	**10-15**	**>15**	
	**(n = 1184)**	**(n = 59)**	**(n = 548)**	**(n = 429)**	**(n = 148)**	
***Gonadotropin total dose (IU)***	2255 ± 1065	2178 ± 863	2205 ± 1099	2283 ± 1070	2390 ± 981	ns
***n. of retrieved oocytes***	8.7 ± 5.2	9.7 ± 5.3	8.5 ± 5.2	8.6 ± 5.1	8.9 ± 5.3	ns
***n. of fertilized oocytes***	5 ± 2.6	5.7 ± 2.7	5 ± 2.6	5 ± 2.6	5.1 ± 2.5	ns
***endometrial thickness (mm)***	10.5 ± 2.6	9.7 ± 2.2	10.0 ± 2.5	11.0 ± 2.5	11.7 ± 3.0	ns
***n. of transferred embryos/ET***	1.9 ± 0.5	1.9 ± 0.5	1.9 ± 0.5	1.9 ± 0.6	1.9 ± 0.6	ns
***Mean embryo score***	8.1 ± 1.8	7.9 ± 1.8	8.2 ± 0.7	8.0 ± 0.8	8.1 ± 0.8	ns
***n. of top scored embryos/ET***	0.5 ± 0.8	0.9 ± 1.4	0.5 ± 0.8	0.5 ± 0.8	0.4 ± 0.7	ns
***n. of pregnancies***	476	24	227	186	39	
***CPR/ET (%)***	40.2	40.7	41.4	43.4	26.4*	0.003
***Implantation rate (IR)***	19.9	19.7	20.9	20.9	12.9*	0.02
***Abortion rate (AR)***	21.4	42.1	17.9	22.0	26.5	ns
***Ongoing pregnancies at 10 W***	374	14	187	145	29	
***Ongoing PR/ET***	31.6	23.7	34.1	33.8	19.6	ns

Patients were subgrouped according to the site at which embryos were transferred (EFD = distance between the hyperecogenic spot and the basal layer of the endometrial fundus).

*significance level of this subgroup vs. all others.

Significantly lower clinical pregnancy and implantation rates were observed in the subgroup with EFD longer than 15 mm than when the embryos were released at less than 15 mm from the fundus (Table 2). Actually the other three subgroups had comparable CPR and IR. IVF outcome was confirmed significantly poorer when the air bubble was observed at more than 15 mm from the fundal endometrial surface even when the analysis was performed considering only patients with normal BMI, or only patients with normal ovarian reserve (basal FSH < 10 IU/L) (not shown). The observed results were confirmed also when the analysis was performed separately for IVF cycles and ICSI cycles (not shown).

When the analysis was performed separately for SET and DET cycles, the results remained significantly poorer for cycles in which the EFD was longer than 15 mm only in case of DET, whereas in case of SET a non-significant trend toward worse results with longer EFD was observed (Table 3).

**Table 3:** Controlled ovarian stimulation and IVF outcome by number of transferred embryos

	**All**	**EFD (mm)**				**p**
		**<5**	**5-9.9**	**10-15**	**>15**	
	**(n = 1184)**	**(n = 59)**	**(n = 548)**	**(n = 429)**	**(n = 148)**	
***n. of pregnancies*** *All*	476	24	227	186	39	
*SET*	49	3	21	21	4	
*DET*	427	21	206	165	35	
***CPR/ET (%) *** *All*	40.2	40.7	41.4	43.4	26.4*	0.003
*SET*	17.2	20.0	18.3	18.7	9.5	ns
*DET*	44.3	42.9	45.3	48.1	30.2*	0.02
***Implantation rate (IR)*** *All*	19.9	19.7	20.9	20.9	12.9*	0.02
*SET*	18.2	22.2	19.6	19.5	9.7	ns
*DET*	20.4	15.6	21.8	21.5	13.8*	0.04
***Abortion rate (AR)*** *All*	21.4	42.1	17.6	22.0	26.5	ns
*SET*	22.4	0	19.0	33.3	0	ns
*DET*	21.3	47.6	17.5	20.6	28.6	ns
***Ongoing pregnancies at 10 W*** *All*	374	14	187	145	29	
*SET*	38	3	17	14	4	
*DET*	336	11	170	131	25	
***Ongoing PR/ET*** *All*	31.6	23.7	34.1	33.8	19.6	ns
*SET*	13.4	20.0	14.8	12.5	9.5	ns
*DET*	37.3	25.0	39.3	41.3	23.6	ns

Patients were subgrouped according to the site at which embryos were transferred (EFD = distance between the hyperecogenic spot and the basal layer of the endometrial fundus) and according to the number of transferred embryos (SET = single embryo transfer; n = 284; DET = double embryo transfer; n = 900).

*significance level of this subgroup vs. 5–9.9 and 10–15.

The abortion rate was noticeably (but not significantly) higher in case of embryo release close to the fundal endometrial surface (EFD <5 mm); as a consequence, in this subgroup of patients the ongoing pregnancy rate at 10 weeks was lower that the one observed for EFD between 5 and 15 mm (Table 2). The abortion rate and the ongoing pregnancy rate were evaluable just for DET because of the insufficient number of observations with SET in some subgroups; the AR was much higher (although not significantly) in case of embryo release nearer than 5 mm from the endometrial mucosa (Table 3).

Overall, the ongoing pregnancy rate was higher when the EFD was between 5 and 15 mm; for EFD above 15 mm the OPR was lower because of a significantly poorer implantation rate, below 5 mm it was lower because a relevantly higher abortion rate (Table 3).

## Discussion

Early studies considering the site of embryo replacement as a variable able to influence IVF success rate suggested that the best results could be obtained when embryos were injected placing the catheter tip as close as possible to the fundal endometrial surface, avoiding to touch the mucosa in order to prevent any endometrial contraction with consequent embryo dislocation [3].

Subsequent studies, however, did not confirm this finding. Differently, they observed a higher PR when the catheter tip was positioned in the middle of the uterine cavity, approximately 15 mm from the fundal endometrium [6]. In another study, an increment of 11% in the PR was reported for every single millimeter, from 0 to 5 mm, of increasing distance of the embryo release site from the fundus of the cavity [15]. Further, a prospective cohort study comparing IVF outcome after upper uterine cavity vs. lower-to-middle uterine cavity ETs showed significantly better results when the embryos were released in the middle of the uterine cavity, approximately from 15 to 20 mm from the fundal endometrium [7]. Also a meta-analysis including three randomized prospective trials [4,5,16] showed a higher pregnancy rate when embryos were replaced in the middle of the uterine cavity than when they were released close to the fundus [13].

Overall, most published reports suggested that replacing embryos between 10 and 20 mm from the fundal endometrium could lead to better IVF outcome than transferring them at other levels of the uterine cavity [6,9,10,13].

Most studies, however, estimated the EFD according to the placement of the catheter tip during embryo injection, without considering that the actual site at which embryos are placed is some mm closer to the uterine fundus than the catheter’s tip. Moreover, a dislocation of the embryos in the few seconds following their release into the uterine cavity is possible, and is not rare. This event has been documented by studies that demonstrated that some technical conditions of embryo transfer are associated with a relevant risk of embryo displacement [17].

In order to better assess the actual site of embryo release, the most recent publications used the US localization of the air bubble that is visible just after embryo release instead of the visualization of the catheter tip [11,12]. A large retrospective study showed that no movement of the air bubble occurs in more than 90% of cases when the catheter is loaded with a low amount of culture medium [18]. In the present study, a low fluid amount (20 μl) was used to load the catheter and a moderate injection speed was used, as recommended to avoid embryo displacement [17,19]: indeed we observed a slight movement of the air bubble (a few mm, for 30–60 seconds) in less than 10% of cases, confirming that the site of embryo release can be estimated by US air bubble detection with high reliability.

A couple of studies using US detection of air bubble localization have been reported to date, but differently from ours they are both retrospective and include much less observations [11,12]. The first investigated the relationship between air bubble position and pregnancy rates in 315 blastocyst transfers, but differently from our study, the EFD was measured with trans-abdominal US [11]. The PR was found to be significantly lower when the EFD was longer than 10 mm [11]. The second study included 409 ETs and failed to demonstrate any significant difference in PR and IR that could be related to the site of embryo release [12]. In fact, both in case of transfer of cleavage stage and blastocyst stage embryos, the mean EFD in IVF cycles that led to conception and in those that did not was the same, and the PR and IR were comparable when the embryos were transferred in the upper or middle third of the uterine cavity [12].

The evidence available to date is, overall, quite conflicting and allows just to state that it is likely that the site of embryo transfer affects IVF outcome; most studies evaluated EFD in an approximate way (looking at the catheter’s tip or using transabdominal US to detect the air bubble) and a well defined EFD range within which embryos must be replaced is not yet been found. Herein we report the first large prospective study in which the impact of EFD on IVF outcome was evaluated using transvaginal US detection of air bubble position, by far the most precise available method to detect embryo position inside the uterine cavity. Our data indicate that optimal PR, IR and ongoing PR may be obtained when embryos are released in the EFD range between 5–15 mm.; if EFD is longer than 15 mm, a significant worsening of IVF outcome occurs, whereas in case of embryos released at less than 5 mm from the fundus, a higher abortion rate occurs, and the ongoing PR at 10 weeks is consequently lower than following ETs in the optimal EFD range. The results that we observed were confirmed when we performed data sub-analyses according to the type of treatment (conventional IVF or ICSI) or the number of embryos transferred (two or one). The overall IVF results observed in our study were similar to those published in the last available European report on IVF outcome CPR/ET of 32.5% for IVF and of 31.9% for ICSI [20]; it must be noted that the results of the SETs included in the present study were particularly disappointing because they were not elective SETs with selected embryos, but just poorly responding patients with only one available embryo. It must be underlined that our observations take into account the embryo morphological quality, the patients’ baseline characteristics (including BMI and ovarian reserve), and the endometrial thickness, and therefore are likely to depend just on the site at which embryos were released.

## Conclusions

In conclusion, our data suggest that an optimal range of EFD exists, and appears to be between 5 and 15 mm, roughly corresponding to the third fourth of the uterine cavity. This is the place where most pregnancies obtained with spontaneous conception may be found during the first US examination at 6–7 weeks, and is likely to be the place where the endometrium displays the best receptivity. Although implantation and pregnancy are not prevented when in vitro produced embryos are transferred at a distance from the fundal endometrial surface shorter than 5 mm or longer than 15 mm, significantly poorer IVF results may be expected, due to a high abortion rate or to a low implantation rate, respectively. The evidence that a defined EFD range allows to obtain optimal results in human IVF suggests that US-guided ET (trans-abdominal or transvaginal) could give some advantage vs. the CTET, provided that the operator inserts the catheter to a depth allowing to deliver embryos within the optimal EFD range.

## Competing interests

The authors declare that they have no competing interests.

## Authors’ contributions

VR, TL, GB and AR conceived the study, participated in its design and coordination, wrote the manuscript. PD performed the statistical analysis. GG and CB critically reviewed the study and helped to draft the manuscript. All authors read and approved the final manuscript.
